# Validation of a sampling method and liquid chromatography mass spectrometry analysis method for measurement of fentanyl and five other illicit drugs

**DOI:** 10.1093/annweh/wxae048

**Published:** 2024-06-11

**Authors:** Matthew Jeronimo, Molly Mastel, Jasleen Gill, Hugh Davies

**Affiliations:** School of Population and Public Health, University of British Columbia, 2206 East Mall, Vancouver, British Columbia, V6T 1Z3, Canada; School of Population and Public Health, University of British Columbia, 2206 East Mall, Vancouver, British Columbia, V6T 1Z3, Canada; School of Population and Public Health, University of British Columbia, 2206 East Mall, Vancouver, British Columbia, V6T 1Z3, Canada; School of Population and Public Health, University of British Columbia, 2206 East Mall, Vancouver, British Columbia, V6T 1Z3, Canada

**Keywords:** air sampling, environmental sampling, fentanyl, illicit drugs, occupational exposure, occupational hygiene

## Abstract

With the increased provision of services by health authorities and community organizations allowing supervised inhalation of illicit substances comes concerns about the potential for secondhand exposure to the substances being used, whether in the adjacent community or to workers at the sites. In order to address community concerns surrounding secondhand illicit substance exposure and better protect harm reduction workers, a validated sampling and LC–MS/MS analysis method was developed for 6 illicit drugs: fentanyl, heroin, methamphetamine, cocaine, etizolam, and bromazolam. It was found that the filter used needed to be silanized to be made more inert and avoid loss of analyte due to degradation. Using the silanized filters, recoveries were good (>90%) and the collected samples were found to be stable at room temperature for 2 wk. The sampling volume validated was up to 960 L. The sensitivity and range of the method make it appropriate for short-term (15 min), full shift (8 h), or environmental sampling.

**What’s Important About This Paper?**

This study developed a validated sampling and analysis method for 6 illicit drugs. This method will facilitate research to describe the magnitude and frequency of secondhand exposure levels to these drugs associated with various jobs and tasks, so as to address community concerns and better protect harm reduction workers, healthcare workers, and others who work with people who use illicit drugs.

## 1. Introduction

The opioid epidemic continues to affect people across North America. Over 14 000 people in British Columbia have died from overdose since the public health emergency was declared in April of 2016 (BC Ministry of Health 2016). The BC death rate remains high, with an average of 5.8 deaths per day, or 45 deaths per 100 000 people in September 2023 (BC Coroners Service 2023). In response to this crisis, British Columbia has taken progressive measures by opening supervised consumption sites (SCS) and overdose prevention sites (OPS), both of which monitor people who are using drugs/substances for overdose and provide rapid response if an overdose occurs (Province of BC 2023). These sites operate under many different configurations (purpose-built structures with filtered heating, ventilation, and air conditioning (HVAC), outdoor tents, etc.) and in a variety of locations, such as standalone sites, within or near housing facilities, at health centers, and/or adjacent to other harm reduction services. There are currently 50 SCS and OPS in British Columbia, and 23 allow smoking of drugs/substances (BCCDC, 2024). With the increased presence of inhalational OPS comes concerns about the potential for secondhand exposure to the substances being used, whether in the adjacent community or to workers at the sites. There is also a desire to increase the number of inhalation sites as smoking is the primary method of consumption (Parent et al. 2021), and the one associated with the most deaths (BC Coroners Service 2023).

In order to address community concerns surrounding secondhand illicit substance exposure, and better protect SCS, OPS, and allied workers, a validated sampling and analysis method was needed for commonly used substances. Our list of substances started with fentanyl; based upon data from the British Columbia Centre on Substance Use, methamphetamine, cocaine, heroin, and etizolam were included. Partway through the study, bromazolam was added to the list to respond to increased presence in the British Columbia drug supply.

The American Conference of Governmental Industrial Hygienists (ACGIH) has recently adopted several Threshold Limit Values (TLV—a health-based exposure limit) for fentanyl (ACGIH 2023). The limits include a surface limit (TLV-SL) of 0.001 mg/100 cm^2^, and 2 limits for fentanyl as inhalable particulate matter: a short-term limit (TLV-STEL) of 0.0002 mg m^–3^ (200 ng m^–3^) and an 8-h time-weighted average (TLV-TWA) of 0.0001 mg m^–3^ (100 ng m^–3^).

There are published sampling and analysis methods for methamphetamine similar to a typical occupational hygiene sampling method, with studies examining airborne concentrations in former clandestine methamphetamine labs, police drug vaults, and simulated contaminated sites (Minnesota Pollution Control Agency 2006; Martyny et al. 2007; VanDyke et al. 2008; Wright et al. 2021). These methods sample airborne methamphetamine onto acid-treated glass fiber filters or a solid-phase microextraction (SPME) sampler and utilize gas chromatography–mass spectrometry for detection. For fentanyl, there are 2 published methods (Van Nimmen and Veulemans 2004; Law et al. 2010) that have performed quantitative air sampling, but they do not agree in their conclusions on filter type, retention, or recovery. There have been 2 Health Hazard Evaluation Reports released by the United States National Institute for Occupational Safety and Health (NIOSH) that offer examples of short-term/occupational sampling (NIOSH 2020a, 2020b), but the analysis was performed at a private laboratory and minimal method information was given. No standardized method has yet been published by NIOSH, or by other organizations.

Environmental sampling for a variety of illicit substances in air (commonly including methamphetamine and/or cocaine) has been performed in many regions across the globe, generally utilizing high-volume samplers (100 to >1000 liters per minute (LPM) flow rate). Data from these studies may be useful to characterize background levels expected to be found in cities. A 2013 review (Pal et al. 2013) gives ranges for a variety of illicit drugs in urban particulate matter; typical values appear to be in the range of tens to hundreds of picograms per cubic meter. High-volume sampling may be necessary to detect these substances in ambient air; however, an easier to deploy method utilizing lower-flow rates (<5 LPM) and smaller sampling heads (25 mm or 37 mm filters) would facilitate more flexibility in occupational sampling, and in environmental sampling in proximity to known use of the substances.

This paper outlines the validation of an air sampling and liquid chromatography–tandem mass spectrometry (LC–MS/MS) analysis method that builds upon previous studies by adding more analytes and clarifying existing knowledge gaps about the optimal sampling media. It is our hope that the availability of this method will increase instances of sampling, thereby providing more data for assessing the potential impacts of environmental or occupational exposure to illicit substances.

## 2. Materials and methods

### 2.1. Chemicals and materials

Fentanyl (CAS 437-38-7), Fentanyl-d5 (CAS 118357-29-2), Heroin (CAS 561-27-3), Heroin-d9(CAS 1338713-49-7), Methamphetamine (CAS 537-46-2), Methamphetamine-d8 (CAS 136765-40-7), Cocaine (CAS -50-36-2), Cocaine-d3 (CAS—138704-14-0), Etizolam (CAS 40054-69-1), and Etizolam-d3 were purchased from Cerilliant (Round Rock, Texas). Fentanyl citrate (CAS 990-73-8), Bromazlam (CAS 71368-80-4), and Bromazolam-d5 were purchased from Cayman Chemical (Ann Arbor, Michigan). Bromazolam was added as an analyte midway through the study at the request of local collaborators. Therefore, not all experiments described in the analytical validation (Section 2.5) have data for bromazolam.

Ammonium formate (LC–MS grade) and Methanol (LC–MS grade) was purchased from Fisher Scientific (Ottawa, Canada). Formic acid (FA) was obtained from Thermo Scientific (Rockford, Illinois), and Sigmacote was purchased from MilliporeSigma (Oakville, Ontario). Whatman quartz fiber QM-A Filters (25 mm, 2.2 μm pore size) and Whatman glass fiber GF-A Filters (25 mm, 1.6 μm pore size) were purchased from MilliporeSigma (Oakville, Ontario). Polyvinyl chloride (PVC) filters (25 mm, 5 μm pore size) and polytetrafluoroethylene (PTFE) filters (25 mm, 2 μm pore size) were purchased from SKC Inc. (Eighty Four, Pennsylvania).

Silanized filters were prepared by placing filters in a shallow crystallization dish and pipetting enough Sigmacote solution to completely cover the filters. The liquid was then swirled for about 1 min before flipping filters with forceps and repeating the swirling of liquid. Filters were then placed on aluminum foil in the fume hood until dry, then were transferred to an oven and baked at 100 °C for 45 min. Filters were then left in a fume hood overnight and the next morning each filter was rinsed 2 times in ultra-pure (18 MΩ cm^-1^) water. Rinsed filters were then placed in 100 °C oven for another 45 min.

### 2.2. Air samplers

Inhalable Fraction and Vapor (IFV) samplers (SKC Inc, Eighty Four, Pennsylvania) and Seven-Hole Samplers (7-HS) (JS-Holdings, Stevenage, United Kingdom) were used for tests requiring simulated sampling. Both collect the “inhalable” size fraction of airborne particulate (International Organization for Standardization 1995). The IFV sampler is operated at 1 LPM and the 7-HS is operated at 2 LPM. GilAir Plus personal sampling pumps (Sensidyne, St. Petersburg, Florida) were used, with the flow rate calibrated using a TSI 4146 mass flow meter (TSI Inc., Shoreview, Minnesota). The IFV sampler also collects gas and vapor phase on a sorbent tube in series behind the filter, but only results from the particulate filter sample are presented in this work.

### 2.3. Solutions

The individual stock solutions were diluted into stock solutions containing all 6 analytes in MeOH with 0.1 % Formic Acid (FA) at concentrations of 400 ng mL^−1^ and 1920 ng mL^−1^. Similarly, a working stock containing the internal standard (IS) compounds was prepared in MeOH with 0.1% FA at a concentration of 5 μg mL^−1^. Calibration standards were prepared in MeOH with 0.1% FA from the working stock. Standards were prepared at nominal concentrations of 0.2, 1, 5, 15, 50, and 80 ng mL^−1^. The extraction solvent used consisted of MeOH with 0.1% FA. All preparation of solutions was carried out in a manner appropriate for handling the compounds used. A risk assessment was performed and safe handling procedures were developed. All stock solutions were stored at −20 °C

### 2.4. Instruments

#### 2.4.1 HPLC conditions.

An Agilent 1200 series HPLC system (Agilent Technologies, Santa Clara, California) consisting of a G1312B binary pump, a G1379B degasser, and a G1367D refrigerated autosampler was employed in this study. One microliter of sample was injected onto a Phenomenex Kinetex Biphenyl column (50 × 4.6 mm, 2.6 μm particle size; Phenomenex, Torrance, California). The column oven was maintained at 40 °C and the mobile phase flow was set at 0.7 mL min^−1^. The mobile phase consisted of (A) water buffered to pH 2.3 with formic acid/ammonium formate and (B) methanol. The timetable was as follows: 60% A from 0 min, 49% A at 1 min, 47% A to 5% A from 2 to 3 min, and 2% A to 0% A from 4 min to 4.5 min, held at 0% A from 4.5 to 5.5 min then back to 60% A from 6.5 min to end of run. Total run time for each sample was 7.5 min.

#### 2.4.2 Mass spectrometry conditions.

An Agilent 6410 triple quadrupole mass spectrometer (Agilent Technologies, Santa Clara, California) was used for detection in this study. The MS was operated in multiple reaction monitoring (MRM) mode and utilized positive electrospray ionization. MS parameters of each drug (Table 1) were individually optimized manually during syringe pump infusion. Nebulizer pressure was 50 psi and capillary voltage was 2,000 V.

**Table 1.** LC–MS/MS parameters for the analysis of target drugs and internal standards by MRM positive ionization mode.

Drug	Precursor ion (m/z)	Product ion (m/z)	Collision energy (eV)	Fragmentor (V)	Mean RT (min)
Cocaine	304.2	182	15	138	2.50
Cocaine-d3	307.2	185.2	15	138	2.50
Etizolam	343.0	313.7	28	125	4.36
Etizolam-d3	346.0	317.0	28	125	4.36
Fentanyl	337.2	188.1	19	135	3.46
Fentanyl-d5	342.2	188.2	19	135	3.46
Heroin	370.2	268.1	27	140	2.20
Heroin-d9	379.2	272.1	27	140	2.20
Methamphetamine	150.2	91.0	15	92	1.37
Methamphetamine-d8	158.2	93.2	15	92	1.30
Bromazolam	353.0	324.5	30	125	4.30
Bromazolam-d5	358.0	329.5	30	125	4.30

Agilent Masshunter Workstation Data Acquisition B.08.02 was used for data acquisition and Agilent Masshunter Quantitative Analysis B.09.00 was used for data processing.

#### 2.5 Analytical validation.

The experiments in the analytical validation portion of this work were based on United States Occupational Safety and Health Administration (OSHA) Validation Guidelines for Air Sampling Methods Utilizing Chromatographic Analysis (OSHA 2010); although the validation method is specific to occupational sampling, the framework is also useful in guiding environmental sampling. In summary, the validation sought to identify if the analytes being sampled could be retained on the sampling media during sampling, that they could be desorbed from the sampling media after sampling, the stability of the collected samples, the stability of the extracted samples, and the sensitivity of the analysis.

#### 2.5.1 Retention efficiency.

The retention efficiency of the selected compounds was tested to determine the optimal filter material for sampling use. The filter media were selected based on previous studies (Van Nimmen and Veulemans 2004; Law et al. 2010). Quartz fiber filters were tested because they were used in a previous fentanyl sampling study (Law et al. 2010) and are commonly used in methods describing high-flow environmental sampling (Cecinato and Balducci 2007; Cecinato et al. 2009a; Postigo et al. 2009; Mastrorianni et al. 2015; López et al. 2022). Glass fiber filters were tested because they were described in a previous fentanyl sampling study (Van Nimmen and Veulemans 2004) as well as multiple methods for methamphetamine (Minnesota Pollution Control Agency 2006; Martyny et al. 2007; VanDyke et al. 2008). PTFE filters were tested because they were used in literature methods for environmental sampling of methamphetamine and cocaine (Cecinato et al. 2009b; Johnson et al. 2023). PVC filters were tested as a cheap, readily available, and commonly used filter in occupational hygiene sampling. Law 2010 found that silanizing the filters was necessary to recover fentanyl from the sampling media; thus, our study also tested silanized glass fiber and quartz fiber filters.

Whatman 25 mm quartz fiber QM-A Filters, Whatman 25 mm glass fiber GF-A Filters, 25 mm PVC filters, 25 mm PTFE filters, silanized Whatman 25 mm QM-A Filters and silanized Whatman 25 mm GF-A Filters were tested. For each test 6 filters were spiked with 50 µL of 1920 ng mL^-1^ mixed drug stock (spiked amount of 96 ng). After being allowed to dry, spiked filters were placed in IFV sampler housing and 3 were attached to sampling pumps calibrated to a flow rate of 1 LPM while 3 were kept as positive controls. For the initial round of tests, a nominal 60 L of ambient air (typically 22C, <30% RH) was drawn through the filters. After the test, filters were transferred to labeled 20 mL scintillation vials. Five milliliters of extraction solution (MeOH with 0.1% FA) were added to each scintillation vial. The vials were then capped and placed on a shaker for 10 min. One milliliter of each solution was then transferred to an auto sampler vial then 10 µL of internal standard solution was added to each vial and analysis was performed using LC–MS/MS. After the initial test the volume of air pumped was increased to 240, 480, and 960 L in subsequent tests. An additional test was conducted at 960 L in a humidified environment (22C, 80% RH).

#### 2.5.2 Extraction efficiency.

To assess the efficiency of extraction recovery 5 filters were spiked with mixed drug interstock solution yielding 4.8 ng, 12 ng, 24 ng, 48 ng, 72 ng, and 96 ng on each filter. These amounts were based upon a range of 0.1 to 2 times the target concentration, with 48 ng being the target amount based upon the recently issued fentanyl TLV-TWA of 100 ng m^–3^ (ACGIH 2023) and 4 h of sampling at 2 LPM or 8 h of sampling at 1 LPM. This range also spans the US Environmental Protection Agency (EPA) Provisional Advisory Level (PAL) 2 and 3 limits for fentanyl (US EPA 2018), assuming 24 h of sampling at 1 or 2 LPM.

After allowing the spikes on the filters to dry, 5 mL of extraction solution (MeOH with 0.1% FA) was added to each scintillation vial. The vials were then capped and placed on a shaker for 10 min. One milliliter of each solution was then transferred to an autosampler vial then 10 µL of internal standard solution was added to each vial and analysis was performed using LC–MS/MS.

#### 2.5.3 Detection limit of analytical procedure (DLAP).

DLAP was measured following the criteria set out in OSHA’s Validation Guidelines for Air Sampling Methods Utilizing Chromatographic analysis (OSHA 2010). Using the estimated signal to noise response of existing calibration samples, a set of 10 analytical standards was prepared for each compound, with the measurement range spanning from below the estimated limit of detection to 10 times above the estimated chromatographic background response. Data obtained from this test was used to establish analytical parameters needed to calculate the DLAP of each drug.

#### 2.5.4 Detection limit of overall procedure (DLOP).

DLOP was measured following the criteria set out in OSHA’s Validation Guidelines for Air Sampling Methods Utilizing Chromatographic (OSHA 2010). Using the DLAP data, a set of ten spiked filter samples (Silanized Whatman 25 mm GF-A) was prepared for each compound, with the measurement range spanning from below the estimated limit of detection to ten times above the estimated chromatographic background response. Each spiked filter was extracted as described above. Data obtained from this test was used to establish analytical parameters needed to calculate the DLOP of each drug.

#### 2.5.5 Tray stability.

Stability of prepared samples in the LC–MS/MS autosampler was tested at 24, 48, and 72 h. Three sets of 4 filters were spiked with 120 µL of 400 ng mL^–1^ interstock solution. Spiked filters were allowed to dry then 5 mL of extraction solvent was added to each scintillation vial containing filters. Vials were then placed on a shaker for 10 min. One milliliter of each solution was then transferred to an autosampler vial then 10 µL of internal standard solution was added to each vial. All 3 sets were analyzed immediately by LC–MS/MS. After being analyzed, both sets remained in the 4 °C autosampler. Two vials from each set had the vial caps which had been punctured by the autosampler needle of the LC–MS/MS replaced. One set was reanalyzed after 24 h, another after 48 h and the remaining set after 72 h. Significance of change in recovery was calculated using repeated measures ANOVA.

#### 2.5.6 Ambient storage stability.

Storage stability of unextracted filters at ambient temperature was tested by spiking 18 filters with 120 µL of 400 ng mL^–1^ interstock solution. Three samples were analyzed by LC–MS/MS on day 1. The remaining samples were left at room temperature in an opaque box and 3 more samples were analyzed on day 5, 3 more on day 8, 3 more on day 11, 3 more on day 14, and the final 3 on day 19.

## 3. Results

### 3.1. Analytical validation

#### Retention efficiency.

**Fig. 1.**
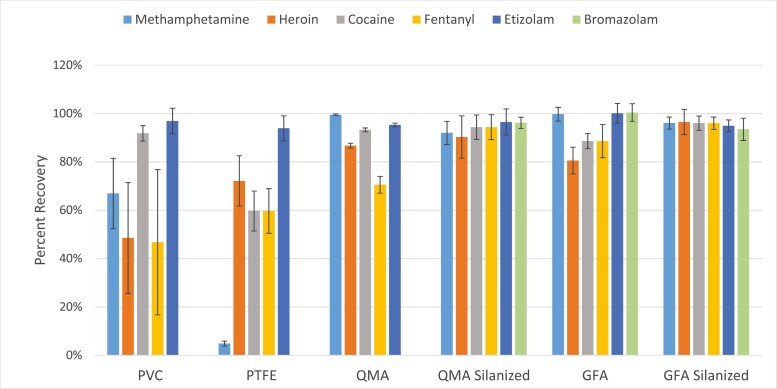
Relative recovery of spiked drugs after 60 L of air sampling versus the type of filter used for sampling. PVC = polyvinyl chloride, QMA = quartz fiber, GFA = glass fiber.

The results from the 60 L retention test on 25 mm QM-A filters, 25 mm GF-A filters, 25 mm PVC filters, 25 mm PTFE filters, silanized 25 mm QM-A filters, and silanized 25 mm GF-A filters are depicted in Fig. 1. In the 60 L test, PVC, PTFE, nonsilanized QM-A, and nonsilanized GF-A filters had variable recoveries, with many filter/drug combinations being below 80% recovery. Of the untreated filters, GF-A had the best retention performance. However, the silanized GF-A and silanized QM-A filter types had retention efficiencies >90% for all drugs.

Following the 60 L test a 240 L test was conducted using only the 3 best-performing filter types: silanized QM-A, GF-A, and silanized GF-A. The GF-A filters performed well for cocaine, methamphetamine (meth), etizolam, and bromazolam but lost approximately 15% of the 2 opioids. The silanized QM-A filters achieved at least 95% recovery for all compounds except Heroin (91%) and the silanized GF-A filters achieved at least 95% recovery for all compounds except Etizolam (91%). The retention test at 480 L was conducted on the silanized QM-A filters and GF-A filters. At this volume, the recoveries on silanized QM-A filters ranged from 89% to 107% and recoveries on GF-A filters ranged from 92% to 101%. Based upon the recoveries as well as the lower cost and higher availability of GF-A filters, silanized GF-A filters were selected for further testing.

The results of the final retention tests challenged with 960 L of air at ambient (approximately 30%) and elevated (80%) relative humidity are shown in Table 2.

**Table 2.** Relative recovery of each drug at different sampled air volumes on silanized GF-A filters. RH = relative humidity.

	Relative recovery (% RSD)					
Liters of air sampled	Meth	Heroin	Cocaine	Fentanyl	Etizolam	Bromazolam
**60 (30% RH)**	96% (2%)	96% (5%)	96% (3%)	96% (3%)	95% (2%)	94% (5%)
**240 (30% RH)**	97% (2%)	95% (2%)	97% (2%)	96% (1%)	95% (3%)	96% (5%)
**480 (30% RH)**	111% (6%)	108% (5%)	106% (6%)	107% (4%)	111% (8%)	113% (7%)
**960 (30% RH)**	97% (3%)	97% (3%)	100% (1%)	99% (1%)	99% (3%)	96% (1%)
**960 (80% RH)**	100% (5%)	93% (4%)	100% (4%)	96% (3%)	99% (3%)	91% (4%)

#### Extraction efficiency.

The extraction efficiency of all 6 drugs on silanized GF-A filters analyzed at each of the 6 specified levels is given in Table 3.

**Table 3.** Extraction efficiency on silanized GF-A filters, by substance and level. “ng” specifies total mass amount spiked on filter. RSD = relative standard deviation.

	Extraction efficiency					
ng	Meth	Heroin	Cocaine	Fentanyl	Etizolam	Bromazolam
96	96%	91%	97%	98%	98%	103%
72	97%	94%	95%	98%	96%	100%
44.8	92%	87%	93%	95%	97%	94%
24	98%	83%	96%	96%	98%	101%
12	91%	85%	91%	93%	93%	97%
4.8	92%	100%	96%	98%	99%	98%
Average	94%	90%	94%	96%	97%	99%
RSD	3%	8%	2%	2%	2%	3%

#### DLAP, DLOP, stability.

The data obtained from the tests described in Sections 2.5.3 and 2.5.4 were used to calculate the Detection Limit of Analytical Procedure (DLAP), Detection Limit of Overall Procedure (DLOP), and Reliable Quantitation Limit (RLQ). All are given in Table 4.

**Table 4.** Detection Limit—Analytical Procedure (DLAP), Detection Limit—Overall Procedure (DLOP), and Reliable Quantitation Limit (RQL) per analyte. *Bromazolam was not added to the method until after DLAP testing. DLAP, DLOP, and RQL are reported in terms of liquid concentration (after filter extraction). RQL is also reported in air, assuming a 4-h sample at 2 LPM.

Analyte	DLAP (ng mL^–1^)	DLOP (ng mL^–1^)	RQL (ng mL^–1^)	RQL—ng m^-3^
Methamphetamine	0.03	0.03	0.11	1.14
Cocaine	0.05	0.04	0.14	1.45
Heroin	0.5	0.26	0.88	9.15
Fentanyl	0.02	0.02	0.05	0.53
Bromazolam	*	0.10	0.34	3.53
Etizolam	0.02	0.06	0.20	2.06

The tray stability test showed that over 24, 48, and 72 h the recovery of all 6 drugs analyzed did not drop or change by more than 10%, which is the criteria given by OSHA Validation Guidelines. This suggests samples may be stored in the autosampler tray for up to 72 h. The actual stable time may be longer than 72 h. Refer to Supplementary Table 1 for all tray stability data.

The storage stability test showed that at ambient temperature the recovery of all 6 drugs analyzed did not change >10% in the first 14 days, which is the criteria given by OSHA Validation Guidelines. This suggests samples are stable before extraction at ambient temperature for at least 2 weeks. The complied data is shown in Supplementary Table 2.

## 4. Discussion

### 4.1. Analytical validation

#### Filter material and sampling conditions.

Existing methods utilizing stock (nonsilanized) glass fiber filters or other noninert filters may exhibit unaccounted-for losses in recovery. At only 60 L of air sampled on a variety of filter types, losses of 10% to 40% were seen for heroin and fentanyl (Fig. 1). For occupational or environmental sampling which would commonly be expected to exceed 60 L (for example, an 8-h sample at 2 LPM would correspond to 960 L), the level of losses may prevent valid interpretation of results. In contrast, the retention efficiency of silanized glass fiber filters was found to be >90% for all 6 compounds at ambient (~30%) humidity and at 80% RH after sampling for 960 L. Silanized glass fiber filters were found to be the most suitable filter media, with satisfactory extraction and recovery characteristics; therefore, these filters are recommended for future sampling. Silanized filters are not available commercially but may be easily prepared following the procedure in Section 2.1. Silanization chemically converts polar and reactive silicate and silianol groups on the glass or quartz filter to nonpolar and nonreactive polysiloxane groups. The loss of opiods on nonsilanized glass and quartz fiber filters was likely due to degradation on the filter. When tests were run with a silica gel sorbent tube following a nonsilanized filter in line, no heroin or fentanyl were found on the sorbent tube, demonstrating that losses in recovery were unlikely to be from volatilization from air flowing over the filter. When the filter media was changed to silanized filters the losses were greatly reduced.

As loaded filters were found to be stable at ambient conditions for 14 day, field samples do not require special storage conditions after sampling. Samples should be sealed, stored away from air and light, and returned to the laboratory for analysis as quickly as possible so that they may be extracted within the 14-day time period.

#### Sensitivity.

The RQL shown in Table 4 range from the single to tens of nanograms per cubic meter (for a 4-h sample). The lack of regulatory OELs or accepted health-based exposure limits makes it difficult to compare the sensitivity of the analysis to the expected sensitivity needed in the field. However, we show that fentanyl has a calculated RQL of 0.5 ng m^–3^ (for a 4 h, 2 LPM sample) which is a factor of 100 below the ACGIH TLV-TWA of 100 ng m^–3^(ACGIH 2023). For a 24 h, 2 LPM sample fentanyl has a calculated RQL of around 0.1 ng m^–3^, which is well below the EPA PAL 2 of 11 ng m^–3^ and the EPA PAL 3 of 3.7 ng m^–3^(US EPA 2018). As the other compounds studied are expected to be less acutely toxic than fentanyl, it is expected that each compound in this method will be quantifiable at and below levels expected to be relevant for occupational and environmental sampling.

#### Study contributions.

The developed method improves upon previously published methods by improving recovery of opioids and allowing for simultaneous analysis of 6 common illicit drugs. This work demonstrates the importance of using silanized filters for analysis of opioids to avoid losses due to chemical degradation.

There is abundant misinformation about the potential for overdose from secondhand inhalation or dermal exposure to substances, specifically fentanyl (Moss et al. 2017; Herman et al. 2020); there have been no confirmed cases of overdose occurring from these types of exposure (Herman et al. 2020). This fear of exposure by workers can harm who use drugs experiencing overdose by delaying their care, and can lead to worsened mental health for first responders (Herman et al. 2020; del Pozo et al. 2022). Further exacerbating this problem is the lack of health information on long-term effects of secondhand exposures. By supporting improved methods of airborne exposure measurement, and thereby providing more data on actual exposure levels associated with various jobs and tasks, we hope to inform discourse around these contentious questions.

Workers in pharmaceutical production, forensic drug laboratories, or who perform clandestine lab cleanups may also be exposed to airborne illicit substances. Voluntary guidelines released by the US EPA (United States Environmental Protection Agency 2021) recommend the use of respirators for cleanup contractors, but only provides guidance for post-remediation clearance air testing. Workers in forensic laboratories have been shown to be exposed to airborne illicit substances (NIOSH 2020a, 2020b). Improved exposure measurement methods will help address the many gaps in knowledge on airborne illicit substances in general, which makes PPE and other controls recommendations limited to expert knowledge rather than peer-reviewed science or specific risk assessments (Basham et al. 2021).

As a new TLV for Fentanyl has recently been introduced (ACGIH 2023), this study, related to improving sampling and analytical methods for the substance, is important to ensure effective use of the new TLV.

## 5. Conclusion

A method has been developed for air monitoring of 6 illicit drugs with an RQL for fentanyl of 0.5 ng m^–3^ which is suitable for monitoring to the proposed TLV. Silanized glass fiber filters were found to be the most suitable sampling media to use. It is recommended that samples be returned to the analysis lab within a week after sampling. Due to the high sensitivity of the analysis method and the stable recovery over the range of sample volumes tested, this method is expected to be suitable for use in “full shift” (nominal 8-h) occupational sampling targeting the fentanyl TLV-TWA of 100 ng m^–3^ and short-term air sampling to collect specific events (such as occupational sampling targeting the fentanyl TLV-STEL of 200 ng m^–3^). For occupational exposure assessment, inhalable size fraction samplers are expected to be used (based upon the airborne TLVs being designated as inhalable), but other samplers may be used as long as the flow rate (1 to 2 LPM) and air volume (60 to 960 L) collected is within the range of sample volumes tested in this work. The method is also expected to be suitable for environmental sampling to determine spatial or temporal distribution of the analyzed substances near inhalational OPS or other sites where there may be airborne substances.

## Supplementary Material

wxae048_suppl_Supplementary_Tables

## Data Availability

The data underlying this article will be shared on reasonable request to the corresponding author.
