# Supplementary material for: Validation of a sampling method and liquid chromatography mass spectrometry analysis method for measurement of fentanyl and five other illicit drugs
Source: Ann Work Expo Health. 2024 Jun 11;68(7):756–64. doi: 10.1093/annweh/wxae048 (PMC11306312; doi:10.1093/annweh/wxae048)
Supplement: wxae048_suppl_Supplementary_Tables [file wxae048_suppl_supplementary_tables.pdf]

Validation of a Sampling Method and Liquid Chromatography Mass Spectrometry Analysis Method for Measurement of Fentanyl and Five Other Illicit Drugs

Matthew Jeronimo[1], Molly Mastel[1], Jasleen Gill[1], Hugh Davies[1]*

[1]School of Population and Public Health, University of British Columbia, Vancouver, British Columbia, Canada

*Corresponding author: Hugh.Davies@ubc.ca

1. SUPPLEMENTAL INFORMATION

Supplementary Table 1 – Tray stability. Percent recovery after storage of extracted samples in instrument vials at 4°C for specified duration.

Tray Stability – Relative Recovery						
**Hours**	Methamphetamine	Heroin	Cocaine	Fentanyl	Etizolam	Bromazolam
**24**	100% (5%)	100% (4%)	100% (2%)	100% (3%)	100% (4%)	100% (3%)
**48**	101% (8%)	93% (6%)	102% (4%)	98% (3%)	95% (4%)	95% (4%)
**72**	103% (2%)	98% (5%)	98% (2%)	96% (3%)	94% (1%)	96% (3%)

Supplementary Table 2 – Ambient storage stability. Percent recovery after storage of filter before extraction at room temperature for specified duration.

Storage before extraction - Relative Recovery						
**Days**	Methamphetamine	Heroin	Cocaine	Fentanyl	Etizolam	Bromazolam
**5**	99% (3%)	101% (4%)	102% (3%)	98% (2%)	97% (5%)	98% (4%)
**8**	95% (5%)	99% (6%)	98% (2%)	93% (3%)	90% (2%)	94% (4%)
**11**	94% (7%)	96% (3%)	102% (6%)	98% (3%)	103% (6%)	99% (3%)
**14**	97% (3%)	97% (2%)	96% (2%)	94% (3%)	91% (4%)	98% (2%)
**19**	88% (4%)	96% (6%)	95% (5%)	93% (6%)	88% (5%)	89% (6%)
